# A review on methods for predicting miRNA–mRNA regulatory modules

**DOI:** 10.1515/jib-2020-0048

**Published:** 2022-04-01

**Authors:** Madhumita Madhumita, Sushmita Paul

**Affiliations:** Department of Bioscience and Bioengineering, Indian Institute of Technology, Jodhpur 342037, Rajasthan, India

**Keywords:** computational methods, miRNA–mRNA regulatory modules, survey

## Abstract

Identification of complex interactions between miRNAs and mRNAs in a regulatory network helps better understand the underlying biological processes. Previously, identification of these interactions was based on sequence-based predicted target binding information. With the advancement in high-throughput omics technologies, miRNA and mRNA expression for the same set of samples are available. This helps develop more efficient and flexible approaches that work by integrating miRNA and mRNA expression profiles with target binding information. Since these integrative approaches of miRNA–mRNA regulatory modules (MRMs) detection is sufficiently able to capture the minute biological details, 26 such algorithms/methods/tools for MRMs identification are comprehensively reviewed in this article. The study covers the significant features underlying every method. Therefore, the methods are classified into eight groups based on mathematical approaches to understand their working and suitability for one’s study. An algorithm could be selected based on the available information with the users and the biological question under investigation.

## 1 Introduction

miRNAs control the regulation of the majority of genes post-transcriptionally. They are short, non-coding RNAs that hybridize with mRNAs and control various biological processes like cell growth and differentiation, apoptosis, oncogenic transformation, and others. miRNAs repress the translation of mRNA transcripts of protein-coding genes either by binding to the transcript or by its degradation. It has been observed that miRNA deregulation plays a significant role in the progression of most human cancers. It has also been associated with the pathogenesis of several multi-factorial and genetic disorders [1]. Therefore, there is a great need to identify regulatory networks comprising miRNAs and their target mRNA transcripts. This can help in exploring their function in specific biological conditions. Several computational techniques have emerged which helps elucidate miRNA function. These techniques can be placed into the following three groups. (1) Methods that help in miRNA target prediction. (2) Methods that help in discovering MRMs. (3) Methods that help in discovering functional MRMs (FMRMs).

The first category comprises methods that deal with the prediction of mRNA targets of miRNAs, based on sequence information [2–5]. The methods in the second category identify modules/groups of co-expressed mRNAs and miRNAs. In contrast, the methods of the last category predict miRNA regulatory networks for the specific biological condition. There is a slight difference between the latter two categories; the purpose of both methods is the same except that FMRMs are condition-specific. FMRMs help in a deeper and critical understanding of underlying biological pathways. It also helps understand the development and prohibition of the pathogenesis of many diseases that MRMs cannot. FMRMs are potentially superior for designing miRNA-based drugs and treatments based on gene therapeutics [6]. The computational approaches/methods that have been designed for the identification of MRMs or FMRMs can be categorized into two major groups: (1) Methods that use only sequence-level information. (2) Methods that integrate sequence level information with the expression profiles of the biomarkers.

**Figure 1:**
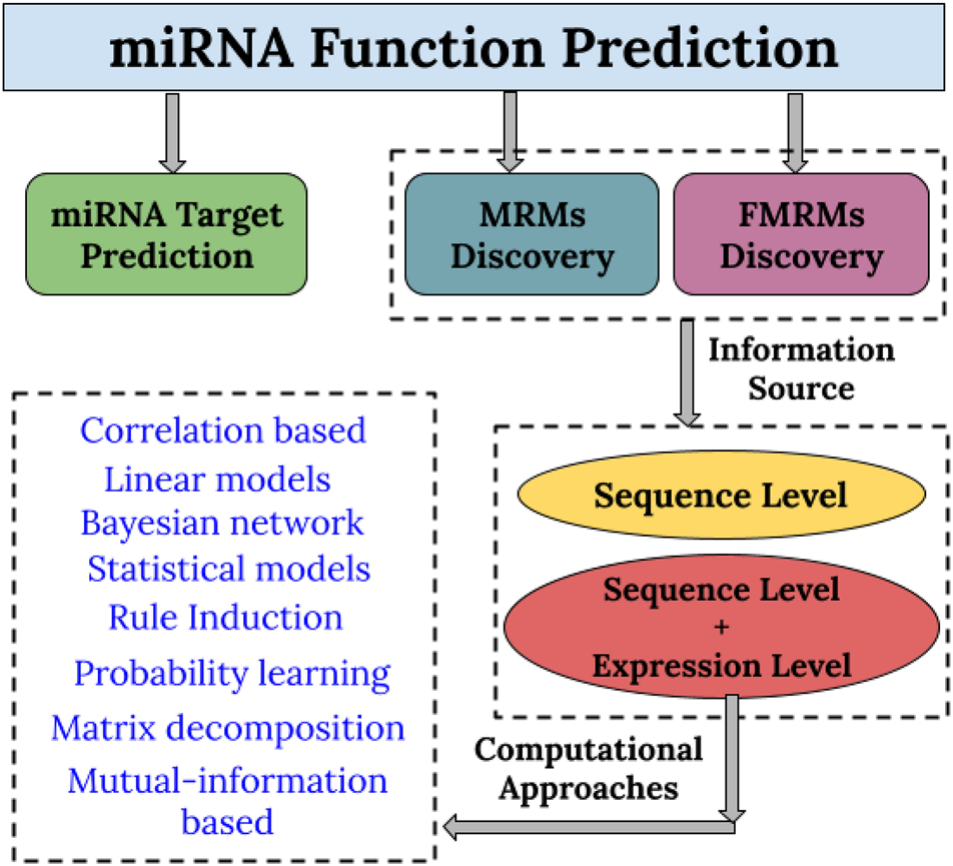
Workflow of the review.

Identifying MRMs/FMRMs is an essential step towards discovering the combinatorial effects of miRNAs and mRNAs of different cellular states. Methods in the first group use sequence similarities to identify miRNAs’ targets are mainly based on seed sequence information and evolutionary conservation. Because of small seed sequences’ availability, these methods are low on sensitivity and predict a large number of false-positive interactions between the two biomarkers [7]. Most of the methods of this group have high computational complexity and are not very helpful in retrieving the functional aspects of the identified modules. In contrast, the second group methods utilize two types of information and integrate them to predict co-expressed groups of miRNAs and mRNAs. The sequence level information also uses these biomarkers’ expression profiles measured across the same set of samples. These methods are often supported by the information derived from sequence-based studies like miRNA-target information, GGI (gene–gene interaction), and others. The dynamic and condition-specific properties of the expression profiles help in better exploration of regulatory modules in comparison to the methods of group one. While most of these approaches use a mere negative correlation to recover some miRNA–mRNA relationships, but fails when it comes to fulfilling the biological context. The problems faced by such straightforward approaches have been overcome by several other powerful and sophisticated approaches that deal with finer details of a biological system. The review attempts to provide an overview of some important and well-known algorithms that use the integrated approach to discover MRMs/FMRMs, the workflow is presented with the help of Figure 1.

## 2 Computational approaches for MRMs identification

Identification of MRMs/FMRMs helps understand miRNA regulatory networks comprising mRNAs and sometimes also the transcription factors (TFs). All the methods developed in this area have a common aim, to detect biologically meaningful regulatory modules. Table 1 represents a list of 26 latest methods. Among the enlisted methods, the methods that are based on integrative approach either make use of a complete set of expression profiles of both the biomarkers derived from a specific biological condition or first identifies differentially expressed biomarkers from the complete set and later on incorporates the sequence-based information to find MRMs/FMRMs from them. All the methods enlisted in Table 1 have been classified into eight groups. They are categorized based on different mathematical approaches used by them to integrate the information from expression profiles to the known target information. (1) Correlation-based approaches use a straightforward way of estimating the correlation between miRNAs and mRNAs. (2) Linear model-based approaches. (3) Bayesian network-based approaches. (4) Statistical model-based approaches use statistical tests to find significant modules. (5) Rule induction approaches use machine learning methods to search for subgroups. (6) Probability-based approaches either use population-based probabilistic learning or probabilistic graphical model to infer regulatory information. (7) Matrix decomposition approaches convert the integrated matrix derived from several types of information into several canonical forms. (8) Mutual information-based approaches. Next, some of the methods from each of the above-mentioned groups are presented in detail.

**Table 1:** List of algorithms/methods/approaches/tools for MRMs identification.

Method	Data sets	Feature	Input data requirement
**Correlation based approach**			
Peng X. et al. [8]^#^	Hepatitis C^*^	Identifies HCV infection-associated MRMs	miRNA and mRNA expression + samples category (diseased and normal)
MAGIA^2^ [9] web-interface	NCI-60^*^	Identifies MRMs by exploring the interplay of miRNAs and TFs in gene/transcripts expression regulation that are involved in mixed regulatory circuits	miRNA and mRNA expression
MirConnX [10] web-interface	GBM^*^	Identifies MRMs that reflects characteristics specific to the data guided by some prior beliefs	miRNA and mRNA expression
Zhang W. et al. [11]^#^	P/MPC	Identifies miRNA–mRNA correlation network modules in tumour subtypes	miRNA and mRNA expression + samples category (tumour subtypes)
Mirsynergy [12] R package	OV, BRCA, and THCA^*^	Detects synergistic MRMs by overlapping neighbourhood expansion	miRNA and mRNA expression + miRNA targets + PPIs
DICORE [13]^#^	EMT, BRCA, and multi-cancer dataset^*^	Detects MRMs by exploring collective group relationship	miRNA and mRNA expression
BCM [14]^#^	BRCA and THCA^*^	Predicts MRMs by iteratively merging the bicliques with the guidance of the GGIs	miRNA and mRNA expression + miRNA targets + GGIs
DmirNet [15]^#^	EMT, BRCA, and MCC^*^	Identifies MRMs by taking advantage of three direct association estimation methods, the bootstrapping and the Ensemble approach based on an inverse-rank-product method.	miRNA and mRNA expression + samples category (diseased and normal)
MIMPFC [16]^#^	EMT, BRCA, and MCC^*^	Identifies MRMs by combining phase-only correlation and improved rough-fuzzy clustering	miRNA and mRNA expression + miRNA targets
CALM [17]^#^	THCA, BRCA, EMT, and OV^*^	Identifies MRMs through integrating the causal interactions and statistical correlations between the miRNAs and their target genes	miRNA and mRNA expression
**Linear model approach**			
Lu Y. et al. [18]^#^	NPC and other tumors^*^	Identifies MRMs based on a Lasso regression model	miRNA and mRNA expression + miRNA targets + samples category (diseased and normal)
Engelmann J.C. and R. Spang, [19] R script	NCI-60^*^	Predicts canonical and non-canonical MRMs	miRNA and mRNA expression + miRNA targets
PIMiM [20]^#^	OV^*^	Discovers MRMs using probabilistic model that combines regression with network information	miRNA and mRNA expression + miRNA targets + PPIs
CoModule [21]^#^	OV^*^	Predicts MRMs in which the miRNAs in each module are expected to present cooperative mechanisms in regulating their targets mRNAs	miRNA and mRNA expression + miRNA targets
**Bayesian network approach**			
SA-Bns [22]^#^	EMT	Discovers MRMs using a splitting and averaging scheme for Bayesian structure learning	miRNA and mRNA expression + miRNA targets + samples category (diseased and normal)
HCTarget [23]^#^	BRCA, PRAD, and MM^*^	Predicts miRNA-target using classical Markov chain Monte Carlo algorithm	miRNA and mRNA expression + miRNA targets
**Statistical approach**			
Liu B. et al. [24]^#^	Mouse mammary dataset	Identifies functional MRMs with correspondence latent Dirichlet allocation	miRNA and mRNA expression + miRNA targets (optional)
Dchip-GemiNI [25] web-interface	LIHC, KIRC, PRAD, LUAD, and GCC^*^	Identifies MRMs in human cancers using TF-miRNA feed-forward loops	miRNA and mRNA expression + TF-gene + TF-miRNA + miRNA targets
Jayaswal V. et al. [26]^#^	Leukemia and MM (Time series data)	Predicts MRMs that contains direct regulation or indirect regulation of mRNAs	miRNA and mRNA expression + miRNA targets
CAPE RNA [27]^#^	Bladder cancer (Urothelial samples)^*^	Predicts MRMs based on individual classification	miRNA and mRNA expression + miRNA targets
**Rule induction approach**			
Tran D. H. et al. [28]^#^	Multiple cancer data sets^*^	Detects MRMs by exploring combinatorial nature of gene regulation	miRNA and mRNA expression + miRNA targets
Song R. et al. [29]^#^	HCV infected humans^*^	Detects MRMs by considering both inverse and positive regulatory relationships between the biomarkers	miRNA and mRNA expression + miRNA targets
Paul S. et al. [30]^#^	COAD^*^	Predicts MRMs using rough hypercuboid based supervised clustering	miRNA and mRNA expression + miRNA targets
**Probability learning approach**			
Joung J.G. et al. [31] available on request	Multiple human cancer^*^	Predicts MRMs via population-based probabilistic learning	miRNA and mRNA expression + miRNA targets
**Matrix decomposition approach**			
SNMNMF [32] Python script	OV^*^	Multiple non-negative matrix factorization based data integration framework for MRMs identification	miRNA and mRNA expression + PPIs + miRNA–mRNA interaction + DNA–protein interaction
**Mutual information based approach**			
RFCM^3^ [33] executable C++ codes	CESC^*^	Identifies MRMs in cervical cancer using MISIM and mutual information	miRNA and mRNA expression + MISIM

The # in the first column indicates that a method is only available as algorithmic steps and no tool/web-interface/scripts are provided. The remaining approaches are freely available in the form of web-interface or scripts that can be modified by the users. The Data sets column represents the system for which the algorithm is developed or the data sets used for bench-marking. An ^*^ represents that the method can be used for any other systems if the required input data is available, whereas no ^*^ means the algorithm is designed precisely for that system (as claimed by the authors). Some of the other terms and abbreviations used in the table are as; NCI-60: A panel of 60 human cancer cell lines from several distinct tissues, GBM: glioblastoma multiforme, P/MPC: primary/metastatic prostate cancer, OV: ovarian cancer, BRCA: breast cancer, THCA: thyroid cancer, EMT: epithelial-mesenchymal transition, MCC: multiclass cancer, NPC: nasopharyngeal cancer, PRAD: prostate adenocarcinoma, LIHC: liver hepatocellular carcinoma, KIRC: kidney renal clear cell carcinoma, PRAD: prostate adenocarcinoma, LUAD: lung adenocarcinoma, GCC: germ cell cancer, MM: multiple myeloma, COAD: colon adenocarcinoma, CESC: cervical cancer, PPIs: protein–protein interactions, GGIs: gene–gene interactions, MISIM: miRNA functional similarity.

### 2.1 Correlation-based approaches

The most simplistic approach to identifying MRMs are whether the biomarkers’ expression is inversely correlated. It means that if a miRNA is up-regulated (or highly expressed) then the target mRNA should be down-regulated [34]. However, some of the studies have also shown that this inverse relationship does not hold true all the time [35–37]. Therefore, the MRMs identified based purely on the inverse regulatory relationship are incomplete in a certain biological context.

#### 2.1.1 A method based on a graph-theoretical approach

**Figure 2:**
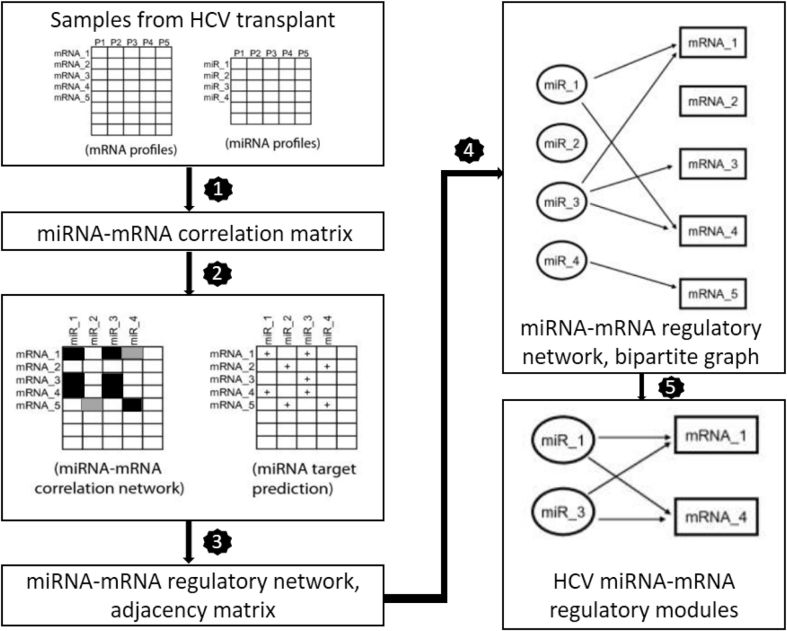
Method based on graph theoretical approach.

In this method, inverse expression relationship between miRNA and mRNA with computationally predicted targets of miRNA have been combined by Peng X. et al. [8], to identify the Hepatitis C virus (HCV) infection-associated MRMs. miRNA regulation in other complex human diseases can also be identified by this method. The regulatory network constructed is in the form of a bipartite graph or a bi-clique, a graph where each vertex of one set (miRNAs) is linked to each vertex of another set (mRNAs) [38]. The method takes the help of Maximal bi-clique enumeration, algorithm [38] and identifies all maximal bi-cliques in the miRNA–mRNA regulatory network. (1) In the first step, the pairwise correlation between each miRNA and mRNA is calculated and stored in a matrix. (2) Next, a binary correlation network is generated from the correlation matrix. A correlation threshold is required for this purpose, which is obtained by calculating false detection rates (FDR) at different correlation values. FDR helps in identifying statistically significant relationships between miRNAs and mRNAs. A threshold is then selected with an overall low FDR as well as it should include the maximum number of highly correlated miRNA–mRNA pairs. (3) In the next step, the above network is superimposed with sequence-based miRNA-target information. A connection is made only if a pair of miRNA and mRNA has a strong negative correlation, and they are also connected in the sequence-based target information. In this way, many-to-many relationships are identified between miRNAs and mRNAs, and a regulatory network is identified. (4) A bipartite graph represents this regulatory network. All the maximal bi-cliques are specified as MRMs. The workflow of this method is presented in Figure 2.

**Figure 3:**
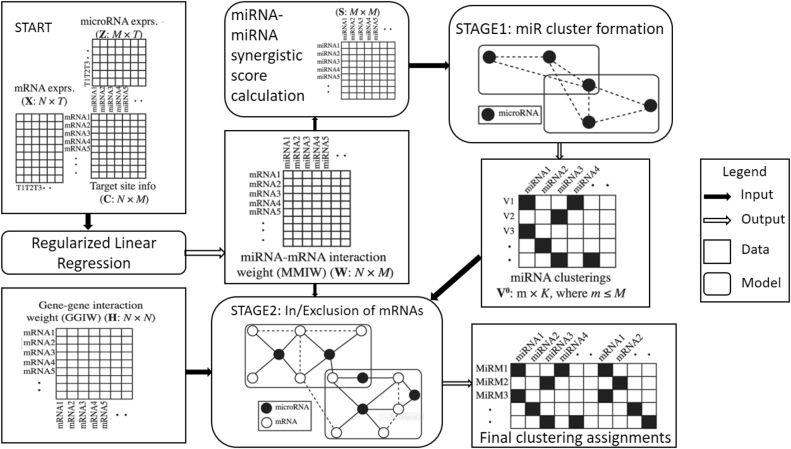
Work-flow for Mirsynergy.

#### 2.1.2 A method that identifies MRMs based on within-class variability

This method is developed by Zhang W. et al. [11], to interpret the genetic regulation of prostate cancer, which is heterogeneous and complicated. The study was to understand the difference in gene regulatory network between primary prostate cancer and metastatic prostate cancer (PPC and MPC). The biological principle governs that miRNAs and mRNAs are differentially expressed across different subtypes or prostate cancer stages. This can also be true for any other cancer or such complex diseases. This within-class variability is primarily due to inherent variation present in the sampled individuals belonging to the same class. This variation is in the form of a molecular genetic mechanism that helps rebuild disease or condition-specific regulatory networks. The steps followed by this method are: (1) miRNA–mRNA correlation matrices are generated, the number of such matrices depends on the number of sub-types under study. Pearson correlation is calculated in a computationally intensive manner for 1000 times. 80% of the samples are randomly selected for every run, and then the mean of 1000 replications are estimated to be the final correlation. (2) The expression correlation matrices are discretized with genes in the rows and miRNAs in the columns. The correlation values are divided into three groups that are denoted by 1 (the top 1% of the positive correlations), −1 (the top 1% of the negative correlations), and 0 (rest of the correlations). (3) The next task is to remove the unwanted miRNAs and mRNAs, to condense the discretized correlation matrices. Therefore, miRNAs (columns) that do not have any recorded relationship with the genes of the biological condition under study are removed. Also, the mRNAs (rows) with less than two non-zero entries are removed. (4) To get a better idea about the complicated interplay among the biomarkers in the correlation matrix and reduce the trivial task of pairwise comparison of the matrix element, a novel method is employed at this step: Fisher’s transformation. The discretized correlation matrix entries are transformed using this method before their decomposition. This helps in an explanation of the results by the standard statistical theory. (5) Then, the correlation matrices are subjected to Hierarchical clustering. This clustering analysis is performed in two ways using Ward’s method and Manhattan distance. (6) miRNA subsets are determined from the clustering result. A couple of two-column topology matrices are generated containing positive and negative connections for each of the miRNA subsets, respectively. (7) Final identification of MRMs is made by dropping off mRNAs having a single connection, either positive or negative.

#### 2.1.3 Mirsynergy

Target site information, GGIs, and miRNA and mRNA expression profiles are altogether used by Mirsynergy [12] for MRMs prediction. The modules discovered by this method contain overlapping mRNAs and miRNAs. The model parameters are predefined, which helps identify a consistent number of modules. Here, the algorithm used for clustering is adapted from ClusterONE [39]. The method tries to construct synergistic MRMs and formulate them as a clustering problem comprising two stages as presented in Figure 3. Stage 1: Only the clustering of miRNAs is considered with the primary aim of the maximization of miRNA–miRNA synergy. This synergistic relationship is directly related to the correlation between miRNAs. Stage 2: MRMs are assigned based on greedily adding or removing genes from them to maximize the synergy score. Gene–gene interaction weights (GGIW) and miRNA–miRNA interaction weights (MMIW) have an equal contribution in defining the synergy score. Incorporation of GGIs information helped in better identification of MRMs, compared to use miRNA–mRNA interaction alone. The advantage of Mirsynergy over other methods are that it uses deterministic formalism and automatically identifies the number of modules. The predefined threshold helps in merging and filtering out of the low-quality clusters. At the same time, it has been shown to improve its computational efficiency by reducing the theoretical bound to only *O*(*M*(*N* + *M*)) from *O*(*K*(*T* + *N* + *M*)^2^) per iteration for *M* miRNAs and *N* mRNAs across *T* samples. Mirsynergy is available at Bioconductor https://bioc.ism.ac.jp/packages/3.0/bioc/html/Mirsynergy.html.

#### 2.1.4 DICORE

The collective group relationship between miRNA and mRNA regulation is the primary idea behind the development of DICORE: The computational framework of Discovering Collective group Relationships [13]. This approach adds quantitative strength information to the identified MRMs. The available data sets are represented with the help of a weighted bipartite graph, and the method searches for a deterministic explanation to the problem of MRMs identification. The MRMs identified by this method have shown significant relevance with the biological condition under study. The step-wise details of DICORE are as follows: (1) At first, the correlation between miRNAs and mRNAs are calculated. The correlation coefficients are stored in a weight matrix *W*, and a weighted bipartite graph represents the interactions. (2) Based on *W*, miRNA–miRNA and mRNA–mRNA collaboration matrices are generated separately. These matrices represent a collaboration score representing the degree of connectivity between the same type of biomarkers. (3) Groups of miRNAs and mRNAs are identified separately. Collaboration scores are used as a similarity measure for generating overlapping clusters. The clustering algorithm used here is adapted from ClusterONE [39]. Clusters having more than 500 and less than 5 mRNAs are discarded. Also, to avert star-shaped networks (networks containing one miRNA and multiple targets), the minimum size for the miRNA group is set to 3. (4) At last, MRMs (COREs) are identified by calculating the canonical correlation [40] between the groups of miRNAs and mRNAs.

#### 2.1.5 BCM: BiCliques merging

BCM [14] is a flexible approach for MRMs discovery that uses expression profiles of both the biomarkers along with miRNA–mRNA target site information and GGIs (gene–gene interactions). Earlier method [8], ends up in identifying a large number of MRMs despite the small size of the network because all the maximal bi-cliques that are statistically significant are considered regulatory modules by them. To overcome this problem, the idea of bi-clique merging is applied by [14] Bi-cliques are merged iteratively in BCM with the proper guidance from GGIs as well as from the overlap present between them. A scoring function facilitates the process of merging. The greedy based merging strategy that helps this method is fast and effective implementation of the merging process. The main steps of the method are as follows: (1) Expression profiles of both the biomarkers and miRNA-target information is utilized to generate a weighted miRNA–mRNA regulatory network. (2) All the maximal bi-cliques are enumerated. The edge weights are not considered while doing so. This gives rise to a large number of entirely connected bipartite miRNA–mRNA sub-graphs. (3) A random shuffling test is performed to assess each of the modules’ statistical significance so that the insignificant ones can be removed from further consideration. (4) The candidate modules are iteratively merged based on their overlaps and GGIs until none of them remains.

#### 2.1.6 DmirNet: direct miRNA–mRNA association network

Reconstruction of several direct regulatory pathways, including direct miRNA–mRNA association networks, is performed by DmirNet [14]. The method tries to solve three major issues related to the other methods proposed to identify MRMs. The first one is traditional correlation-based methods to find correlations between biomarkers based on their expression. These methods may represent various inaccurate connections or overestimate edge weights because of transitive information flow among direct associations. The next issue is related to the dimensionality of the study data sets. The availability of high dimensional low sample size data sets creates difficulty in calculating reliable and accurate empirical correlations between all pairs of expression profiles. The last issue is the variation in the performance of these methods across different data sets. More reliable models are needed to tackle the above-mentioned shortcomings that can show optimal or sub-optimal performance across different data sets. The steps followed by DmirNet: (1) In the first step, irrelevant miRNAs and mRNAs are removed so that only the active miRNA–mRNA interactions remain. (2) The biomarkers, which pass the criteria to be called differentially expressed, are then integrated and scaled. (3) Next, the integrated expression profile is subjected to three different direct correlation inference methods that are based on bootstrapping strategy. The method used is Partial correlation [41], Sparse Partial correlation (SPACE) [42] and Network deconvolution [43]. (4) Each of these methods generates a matrix based on a direct correlation model from the expression profiles containing all combinations of miRNAs and mRNAs. (5) Integrating the relationship derived between all the biomarkers from different methods is done to generate the final direct correlation matrix using a rank-based aggregation method. (6) The above step’s output is finally used to regenerate a direct miRNA–mRNA association network by assigning threshold to the weights. In a nutshell, it can be said that DmirNet tries to improve the MRMs identification approach by taking complete advantage of bootstrapping, inverse-rank-product-based ensemble approach, and the three direct associations estimation methods.

#### 2.1.7 MIMPFC

MIMPFC has efficiently combined improved Rough Fuzzy Clustering (IRFC) and Phase Only Correlation (POC) [44] to identify MRMs. The principle behind this method’s working is inspired by the relationships of the collective group [45], and DICORE [13]. DICORE explores the interacting strength between groups of miRNAs and mRNAs instead of their individual strength. The uncertainty present in the data is penalized. This penalty term is based on an assumption of undiscovered interaction between the two biomarkers, which leads to a biased result. Therefore, to avoid such uncertainties, MIMPFC combines POC and IRFC for clustering interacting groups of miRNAs and mRNAs. Following are the steps of MIMPFC [16]: (1) In the first step, the interaction matrix W is generated. POC is applied to find pair-wise interaction between miRNAs and mRNAs. The expression of these biomarkers across a common set of biomarkers is utilized for this purpose. (2) Both the information sources (miRNA and mRNA expression) are used individually to generate similarity fuzzy score matrices *P* and *Q*, respectively. (3) miRNA and mRNA classes are inferred by using IRFC. To avoid removing the potential biomarkers, the classes having less than 3 miRNAs and less than 5 mRNAs are re-clustered. (4) At last, canonical correlation [40] is applied to identify interacting miRNA and mRNA classes.

#### 2.1.8 CALM: causal regulatory modules

The causal relationships between miRNAs and their target genes have been explored in [17] for MRMs discovery, which has been neglected by the other methods. Statistical correlation and causal interaction between both the biomarkers are simultaneously explored by CALM to identify biologically significant MRMs. This integration helps avoiding incorrect regulations. Following steps are taken by this method to identify MRMs: (1) Intervention calculus when the DAG is absent (IDA) strategy [46, 47] has been used to build causal interaction between miRNAs and mRNAs. The interactions are represented in the form of a Directed Acyclic Graph (DAG). The nodes of this graph represent miRNAs and mRNAs, and the edges represent the interaction. The steps of IDA strategy applied here are: (a) PC (Peter and Clark) algorithm [48], is used to explore the causal interaction among the biomarkers based on their expression. To incorporate sparsity in the matrix, the PC algorithm is modified by reducing the value of alpha. Then the causal interaction between miRNAs and mRNAs are calculated by using the do-calculus [49] strategy. (b) In order to avoid the problem of over fitting, here a bootstrapping strategy is applied. (c) At the last step, the significance of the identified interactions is evaluated based on a two-step KS static. Interactions having a *p*-value lesser than 0.05 are only considered. (2) For the estimation of miRNA–miRNA regulatory interactions, functional similarity between their target genes is considered. R package GO-Semsim [50] is used to estimate these interactions. (3) MRMs are identified by greedily adding or removing the target gene to maximize the modularity score similar as done in ClusterONE algorithm [39].

### 2.2 Linear model based approaches

Most of the correlation-based approaches depend on calculating pairwise relationships between the two biomarkers. However, several studies have shown that multiple miRNAs can regulate the expression of a single mRNA. Therefore, linear model-based approaches identify the combinatorial effect of multiple miRNAs on a single mRNA.

#### 2.2.1 PIMiM

PIMiM: Protein Interaction-based MicroRNA Modules [20] is a regression-based probabilistic method. It works by integrating interactions, expression profiles, and sequence data and identifies mRNA modules that are regulated by a small set of miRNAs. PIMiM is applied to the cancer data. It shows that incorporating PPIs data and accurate modeling of coordinated miRNA–mRNA interactions helps the method accurately identify the regulatory modules. The steps of PIMiM are as follows: (1) Four matrices are taken as input by this method, two of the expression matrices and two of the weighted adjacency matrices. The adjacency matrices Ω and *ϕ* contain the protein interactions and the predicted miRNA–mRNA interactions from sequence data. (2) The number of modules (*K*) to be identified is predefined. This method’s primary aim is to calculate the propensity for every miRNA and mRNA that represents their belongingness in module k. The membership parameters, u_*ik*_ and v_*jk*_ denotes the belongingness. Where *u*_*ik*_ and *v*_*jk*_ > = 0. (3) The miRNA regulators are specified, and the weights for the probabilistic regression model are learned based on two assumptions. According to the first assumption, all the mRNAs of a module are targets of the miRNAs if and only if they are also the predicted targets. The second assumption suggests aggregation of down-regulated weights is present across all the modules. These assumptions make this method different from all the other methods that use the probabilistic regression model. (4) To incorporate the interaction data (Ω and *ϕ*), a function is formulated that works by rewarding strong connectivity between the predicted miRNA targets if they belong to the same module. The model tunes the contribution of positive and negative interactions by the *α* and *β* parameters, respectively, and *σ*(.) is the logistic sigmoid function. The interaction probability between miRNA and an mRNA and between two genes/proteins is directly proportional to the chance that the interacting biomarkers will lie in the same module. (5) Finally, the log-likelihood optimization function is minimized to find the MRMs. This function has three components. The first one estimates the interaction between miRNAs and mRNAs based on their expression. The second and third component deals with rewarding predicted miRNA targets and PPINs, respectively. (6) The convex nature of this log-likelihood function ends up in finding several local minima. Therefore, to restrict multiple solutions, two sets of *l*_1_ norm constraints and two different regularization parameters (*C*_1_ and *C*_2_) are added. The probabilistic model developed here integrates network information with regression to discover modules, which is very different from other probabilistic model-based approaches. To combine the data from multiple conditions, a new iterative learning procedure is developed that learns the parameters of the proposed model and helps decipher condition-specific regulation of miRNAs with the help of MRMs identification.

#### 2.2.2 CoModule

Like the other methods, CoModule [21] also tries to identify overlapping MRMs by integrating diverse data sets. This is a cluster-based computational method whose ultimate goal is to detect such MRMs, in which each miRNA represents a cooperative mechanism in regulating their target mRNAs. To fulfill this purpose, at first CoModule clusters the miRNAs based on a similar expression. A rough set clustering approach is applied for this purpose. Once a credible amount of miRNA clusters has been obtained, regulators’ targets are added naturally into the corresponding clusters to produce the final MRMs. The precise miRNA–mRNA interactions are reconstructed by using the LASSO regression model, which considers expression profiles of both the biomarkers and sequence-based predicted target sites information.

### 2.3 Bayesian network based approach

Past studies have shown effective use of the Bayesian network, a probabilistic graphical model for identifying complex gene-networks [51]. Keeping this in view, similar approaches have also been developed and applied to study and understand the regulatory information between the two biomarkers (miRNAs and mRNAs) by modeling the whole miRNA–mRNA regulatory network.

#### 2.3.1 SA-BNs: splitting and averaging scheme for Bayesian networks

The method developed by Liu B. et al. [22], uses the strategy of splitting-averaging to learn the Bayesian network so that the complex interaction between miRNAs and mRNAs in different physiological conditions can be modeled with maximum accuracy. SA-BNs first identify a set of differentially expressed biomarkers between the multiple conditions under study. Welch *t*-test with 10,000 times permutation (*p*-value < 0.05, adjusted by Benjamini and Hochberg (BH) method) is used for this purpose. Following are the steps: (1) After the extraction of differentially expressed biomarkers, the expression profiles are split according to the categories of samples. (2) Discretization is done as a standardization means for the data as they are derived from a different platform. (3) Next, the dependency of the biomarkers is estimated from the discretized expression profiles for the respective sample categories by learning a Bayesian network structure. At this step, to avoid false discoveries, miRNA-target information is used. (4) In the final stage, all the Bayesian networks learned for the different sample categories are merged by taking an average. Thus an overall miRNA–mRNA interaction network is generated. The method takes the help of bootstrapping [52], that is, re-sampling with a replacement for robust interference. This helps it in dealing with a small sample size of miRNA or mRNA expression profile. Also, to overcome the computationally consuming task of Bayesian network learning, it utilizes the concept of constraint-based space searching. Here, the constraints are in the form of domain knowledge. This method outperforms those methods that use a normal Bayesian network for finding MRMs.

### 2.4 Statistical approaches

The application of statistical methods in extracting knowledge from multiple information sources have been widely explored. These methods use very few assumptions and parameters and help develop robust models that can identify MRMs having significant biological relevance [25–27].

#### 2.4.1 MRMs identification by integrating guided and unguided clustering

The method proposed in [26] has two steps; first, it identifies the miRNA and mRNA clusters separately. Later, association between these two clusters are estimated, and the clusters having statistically significant associations are reported as potential MRMs. The method works as: (1) miRNAs and mRNAs are clustered separately by unguided clustering (Clust_UN_) and also by guided clustering (Clust_GD_). Changes in expression profiles are not considered in Clust_UN_. (2) Clusters returned by both the methods for both the biomarkers are evaluated separately to find statistically significant miRNA and mRNA clusters. A non-parametric bootstrap test is used on enrichment analysis to do so. (3) Next, a statistically significant association between miRNA–mRNA pairs are determined. A pair is only considered associated only if the computational prediction accords with the change in expression of miRNAs and mRNAs. A linear model tests the latter condition. The method helps in the identification of two types of mRNA clusters. One that is co-regulated by a set of miRNAs and others regulated by just a single miRNA. miRNAs that share very few co-targets belongs to a cluster of size one. The method’s detection of MRMs may vary based on the input matrix that contains the information about computationally derived miRNA–gene targets. As observed by Jayaswal V. et al. for the time-course data set, the modules detected by using the combination of miRanda [53], TargetScan [54], PicTar [5] and miRGen [55] were different from the modules detected by using TargetMiner [56].

#### 2.4.2 Corr-LDA inspired FMRMs identification method

The sample matched miRNA and mRNA expression data, profiled across multiple classes of conditions or tissues give ample opportunity to systematically investigate plausible FMRMs in various biological conditions, even without considering the target binding information. Several studies have shown that the methods that do not utilize the already known miRNA–target interactions may help reduce biases [7, 54, 57]. The method used by Liu B. et al. [24], for the identification of FMRMs is inspired by Corr-LDA (Correspondence Latent Dirichlet allocation) strategy [58]. Corr-LDA’s concept is successful for the automatic annotation of images with their captions. For FMRMs discovery, every module is considered as an independent group linked to a latent function. The method then models the functional modules by exploring latent random variables, which act as a connecting link between miRNAs and mRNAs.

#### 2.4.3 CAPE RNA

CAPE RNA: Classification based Analysis of Paired Expression data of RNA [27] helps in capturing altered miRNA–mRNA regulation between different biological conditions. It identifies the altered regulation between tissue samples without having prior information about the stratification of the groups. When applied to the expression data of normal and cancerous samples, the method could capture differentially regulated miRNA–gene interactions. The steps of CAPE RNA are as follows: (1) miRNA and mRNA expression profiles are first normalized and partitioned into three sets: “high,” “medium,” and “low,” based on their respective expression values. (2) Filtration of interaction states is performed. miRNA and mRNA probes having a score greater than a certain threshold (*θ*_score_ = *t*|E|) are only considered for the further steps. This helps in the identification of sets of biomarkers having somewhat similar expressions. At the same time, both sets together should cover the whole dataset with minimal overlap. (3) Classification of every miRNA–mRNA interaction for each of the samples is performed. At this stage, sequence-based interaction information is also considered. (4) A metric called Jaccard-index is then considered to estimate the overlap between the experimental and the expected groups. This calculation is based on the assumption that mRNAs regulated by a specific miRNA are up-regulated in one group and down-regulated in the other one. This helps in searching for all the differentially regulated miRNA–mRNA interactions. (5) At last, the obtained differentially regulated interactions are merged based on a negative correlation between the interacting species to make a final selection of MRMs. The classification of gene expression data performed by this method is based on certain biological assumptions. This helps the method in reducing the information content to a greater extent. Also, CAPE RNA does not use statistical tests like *t*-test or mean/median comparisons for different groups; this helps CAPE RNA reduce errors occurring due to outliers and prevents the underestimation of the regulation of a single sample. Therefore, the combined set of miRNA–mRNA interaction states are examined.

### 2.5 Rule induction based approaches

In a rule induction technique, rules are generated from a set of input variables with information theory calculation. The rules are generated so that only those input variables get selected that are most relevant to the values of output variables. The identification of MRMs rules is generated from multiple information sources and then tried to be integrated into a meaningful fashion [28–30].

#### 2.5.1 Confidence and coverage based rule induction method

The method used in [28] to discover MRMs is based on the rule induction approach. Such machine learning approaches have been successfully applied in subgroup discovery. There are mainly three ways for inducing rules from data: exhaustive search, separate and conquer, and divide and conquer [59]. In this method set of miRNA–mRNA regulatory rules are produced using the CN2-SD rule induction system [60]. This system is an improvement to the CN2 approach that uses separate and conquer strategy [61]. Following are the steps of the method: (1) At first, the correlation between the first gene and the rest of the genes is calculated. (2) Based on the correlation threshold, the gene set is divided into two classes, *similarity*, and *dissimilarity*. (3) The interaction information from the miRNA-target binding information table is now taken into consideration. Based on these interactions, a column indicating class is appended to the miRNA binding information table. After this addition, the table becomes a regulatory decision table for the current gene. (4) Next, regulatory rules are defined by using the CN2-SD rule induction system. (5) Filtration of the insignificant rules is done. Rules which contain miRNAs with highly correlated expression profiles are only considered for generating potential MRMs. (6) The above process is repeated for all the genes one by one in the gene expression profile table.

#### 2.5.2 Supervised Clustering based rule generation method

RH-SAC (Rough Hyper-cuboid based Supervised Clustering) deciphers the regulatory interactions present between multiple miRNAs and mRNAs expressed in the patients suffering from colorectal cancer [30]. The objective of this method is to discover groups of miRNAs and mRNAs that are functionally similar. Also, the coherent expression of such groups should classify the clinical outcomes. The method also calculates metrics like similarity/redundancy to identify the relationship between the selected miRNAs and mRNAs. The concept of RH-SAC [62] helps this method efficiently handle those uncertainties that arise during the expression data analysis. Next, the steps of this method are discussed. (1) First, the RH-SAC approach of clustering is applied to miRNA expression data to identify miRNA clusters/rules. When employed to the SVM classifier, the average expression of these clusters was able to classify the samples. Two cross-validation methods are also used to check on classification accuracy, namely leave-one-out-cross validation and 10-fold cross-validation. (2) Next, for each of the miRNA rules, target mRNAs are assigned to them. The experimentally validated miRNA-targets are used for this purpose. (3) The reduced set is now subjected to the RH-SAC algorithm to generate mRNA rules. It helps in searching for a group of functionally similar and differentially expressed mRNAs. (4) Finally, the miRNA and respective mRNA rules are merged to generate MRMs. This approach can identify biologically relevant MRMs in different conditions and is suggested to be used in larger sample groups. The method can examine sub-type-specific unique miRNA–mRNA interactions.

#### 2.5.3 Connected discriminatory rules generation method

The method proposed in [29] focuses on the identification of both positive and inverse regulatory relationships from miRNA and mRNA expressions profiled on the same set of Hepatitis C virus-affected tissue samples. The method can also be applied for the identification of such relationships in other complex human diseases. The method is composed of two sequential steps that use a “change-to-change” approach to identify interrelated discriminatory rules, and the steps are as follows: (1) At first, miRNA rules are generated. The rules contain a set of differentially expressed miRNAs with a frequency of 100%. Next, these biomarkers are ranked using gain-ratio criterion [63, 64] through Weka 3.6 software package (http://www.cs.waikato.ac.nz/ml/weka/). The committee tree approach is used to detect 100%-frequency. (2) Public data is searched to identify predicted mRNA targets for every miRNA in each rule and a set of mRNA for each miRNA rule is selected. (3) Now, on this reduced set of mRNAs, data mining techniques are applied to identify mRNA rules for 100% frequency. (4) Finally, the grouping of all mRNA rules of 100% frequency for each miRNA rule is done to identify MRMs. These modules are represented by a bipartite graph, where both of the biomarkers are kept in their respective parties. Positively regulated miRNA–mRNA pairs and inverse expression relationships exist in many-to-many regulatory modules; this biological principle makes this method unique in its implementation and MRMs discovery.

### 2.6 Probability learning based approach

The techniques that use the probability learning approach for MRMs identification try to predict the probability of certain miRNA–mRNA interactions. Multiple information sources are used for such predictions under different biological conditions.

#### 2.6.1 Population based probabilistic method

The method proposed in [31] identifies coherent MRMs based on the assumption that they share similar biological functions. Along with the expression profiles of miRNAs and mRNAs, the predicted miRNA–mRNA interactions are also used. Both miRNA and mRNA expression profiles have different scales and variations; merely combining them will lead to a poor result. Therefore, a population-based probability learning method is used, built on co-evolutionary learning and estimation-of-distribution algorithms [65–67]. This method helps in combing multiple data sets more effectively. The steps are as follows: (1) In the learning process, random populations of miRNAs and mRNAs are selected from each of the expression profiles. Each population is assigned a probability vector. (2) The Fitness measurement binding score is calculated for each individual in both populations. (3) The best individual is selected based on fitness scores. Incorporation of this strategy imbibes a co-evolutionary learning effect and helps find a complete solution. (4) The probability vectors for the two populations are updated. Two parameters (*δ* *m* ∈ (0,1] and *δ* *t* ∈ (0,1]) are defined to control the update rate. When these parameters attain a value closer to zero, the probability vector assigned at this stage is highly dependent on the prior probabilities. (5) The present probability distribution of both the biomarkers is updated, and new populations are generated. (6) The above steps of updating the probability distribution and generating the new populations are repeated until the maximum number of generations is reached. The cooperative fitting of two groups of miRNA and mRNAs to the best solution finds MRMs with significantly high fitness scores. This method is similar to the bi-clustering method, where clustering of rows and columns is done simultaneously on a two-dimensional matrix. Though bi-clustering has been widely used to solve several biological issues [68], it has one shortcoming that it requires the prior setting of several fitness parameters. Hence, there is a need to reduce the number of parameters. Also, the reduction should maintain an implicit balance between several objectives. Therefore, designing a multi-objective optimization technique can tackle this problem.

### 2.7 Matrix decomposition based approach

The decomposition of multivariate data has been successfully attained by non-negative matrix factorization (NMF). For the integration of multiple information sources, the NMF framework has been used multiple times in a regularized manner.

#### 2.7.1 SNMNMF: sparse network-regularized multiple negative matrix factorization

Effective integration of heterogeneous data by Zhang et al. helps in the prediction of miRNA–gene regulatory co-modules [32]. The integration is done in a regularized manner to capture information from different sources like the expression profiles of both the biomarkers along with miRNA–target interactions and GGIs. Earlier attempt to jointly analyze expression profiles in a multiple NMF framework could not attain a modular solution [69]. Therefore, to overcome this shortcoming, sparsity penalties are applied to the variables in this method called SNMNMF. This penalty component also helps enhance the signal-to-noise separation and, at the same time, perk up the interpretability of the obtained MRMs. In a basic NMF problem, expecting to attain a global minimum with a standard optimization algorithm is very unrealistic. Therefore, to attain a local minimum, the process of matrix decomposition is iteratively updated by the algorithm SNMNMF. To attain this local minimum, an objective function is framed that has three elements. Each of these three elements has a specific task. The first one deals with the two non-negative matrices, namely, *X*_1_ and *X*_2_, representing two expression matrices. The second and third elements take care of interaction constraints occurring due to GGIs and miRNA–target interactions. This objective function is then optimized to attain a joint decomposition of both the matrices *X*_1_ and *X*_2_. This decomposition helps in obtaining MRMs. The steps followed by SNMNMF are: (1) These are the required inputs for the algorithm: (a) matrices *X*_1_ and *X*_2_ (b) a network containing information about DNA–protein and protein–protein interactions called as GGIs and is represented by the matrix, *A* (c) another matrix *B*, that contains information about miRNA–target interactions. (2) A common basis matrix, namely *W* and two coefficient matrices, namely, *H*_1_ and *H*_2_ are obtained by simultaneous factorization of *X*_1_ and *X*_2_ respectively. Also, the information content of matrix, *A* and *B* are utilized to incorporate the network’s regularized constraints. (3) Information about miRNA–gene regulatory co-modules is derived from the decomposed matrix component. The basis of identification of co-modules is shared components (a column in *W*) with significant association values in the corresponding rows of *H*_1_ and *H*_2_. The python scripts for SNMNMF is available at http://nimfa.biolab.si/nimfa.methods.factorization.snmnmf.html.

### 2.8 Mutual information based approach

This section describes a method that helps in the identification of FMRMs in Cervical cancer. The method utilizes the expression variability of miRNA and mRNA across a common set of samples to identify star-shaped modules in the beginning, having one miRNA and up to fifty mRNAs. Later these star-shaped modules are merged to get biologically significant modules based on MISIM (miRNA functional similarity) information [70]. Next, the method is described in detail.

#### 2.8.1 Relevant and functionally consistent miRNA–mRNA modules

Relevant and Functionally Consistent miRNA–mRNA Modules (RFCM^3^) [33] uses Mutual Information (MI) to identify regulatory modules containing multiple biomarkers of both types. A two-stage approach, which is used to derive relevant and functionally consistent MRMs are described further.

Stage 1: *Identification of Star Shaped Modules* – At first star-shaped modules containing one miRNA and a maximum of fifty mRNAs are generated. The expression value of mRNAs and miRNAs are first discretized by using the discretization method mentioned in [71]. Then, the relevance between a miRNA and all the mRNAs is calculated using MI. The most relevant mRNA is selected, having the highest value of MI. This selection makes the most relevant mRNA as a module member. The above steps are iterated till the required number of mRNAs get identified for a module. The selection of the next mRNA form the remaining mRNAs is only done if it maximizes the criteria: $0.5*\hat{f}\left(Y_{j}\;X\right)+0.5*\frac{1}{\left|\Theta \right|}\sum_{Y_{i}\in \Theta }\tilde{f}\left(Y_{i},Y_{j}\right)$ . Here, $\hat{f}\left(Y_{j},X\right)$ represents relevance: MI between miRNA and mRNA, and $\tilde{f}\left(Y_{i},Y_{j}\right)$ represents functional similarity: MI between two mRNAs. Also, mRNAs having functional similarity lesser than 0.15 with the already selected mRNAs in a module is not considered. This stage generates *m* number of star-shaped modules, where *m* is the number of miRNAs present in miRNA expression matrix taken as input.

Stage 2: *Infusion of miRNA–miRNA functional similarity information* – In this step, star-shaped modules are merged into MRMs containing multiple miRNAs and mRNAs that are biologically relevant to cervical cancer. MISIM similarity is used for this. In the MISIM matrix, pairwise functional similarity between the miRNAs related to cervical cancer is represented through normalized scores between 0 and 1. The higher the score stronger is the interaction, whereas a 0 means no interaction at all. These functional similarities are calculated based on the assumption that a group of functionally related miRNAs is most probably associated with the same kind of diseases, and DAGs can represent these associations. RFCM^3^ varies the functional similarity score from 0.7 to 1 for merging multiple star-shaped modules. If the MISIM similarity score between the miRNAs of two modules is greater than these cut-offs, they are merged into one module with multiple miRNAs and mRNAs. The obtained modules are passed to pathway enrichment analysis using DAVID annotation tool [72, 73], and a quantitative index named KPES (Kegg pathway enrichment score) is calculated to capture the biological relativity of these modules. Similarity value at which the modules have maximum KPES is considered as the optimal cut-off score and the modules as the final MRMs for cervical cancer. RFCM^3^ can be applied to any disease-specific miRNA and mRNA expression data where MISIM similarity is available. The executable codes for RFCM^3^ is available at http://home.iitj.ac.in/`~sushmitapaul/CBL/softwares.html.

## 3 Recommendation for the users

**Figure 4:**
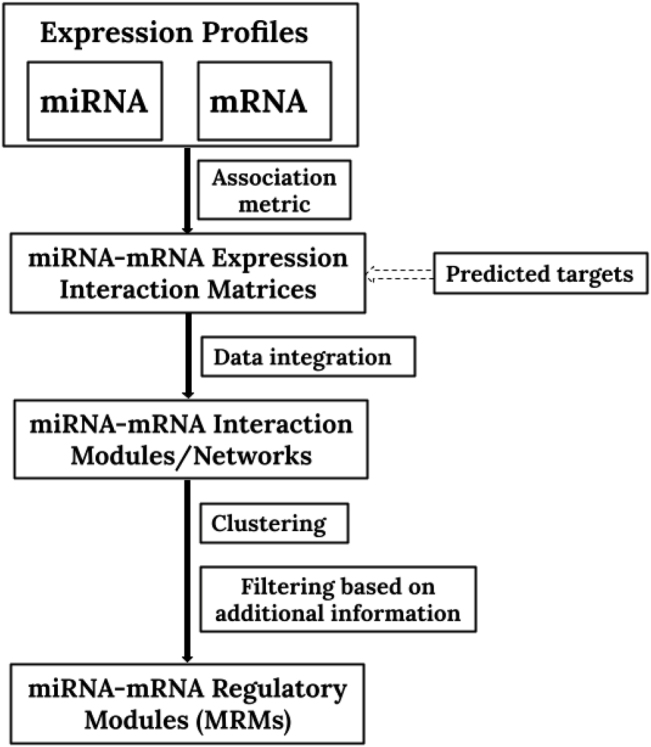
Schematic portrayal for the identification of MRMs.

A schematic portrayal for the identification of MRMs is presented in Figure 4. At first, the miRNA–mRNA interaction table/matrix is generated by integrating expression profiles using an association metric like correlation/MI or others. Sometimes miRNA-target information is also included. Then, some clustering technique is used to cluster both the rows and columns simultaneously (miRNA and mRNA) to generate MRMs. Later, a filtration step is added to derive the significant modules. Several researchers have regularly explored this general platform, and the integration, clustering, and filtration steps have been modified to get biologically relevant MRMs. Some methods even incorporate the GGI information at the filtration or integration step. Some methods conduct integration and later steps on differentially expressed biomarkers. Another group uses rule induction; they cluster miRNAs first and then add mRNA by incorporating target information. A different set of algorithms is also available, which only uses expression data to avoid bias. These methods generate the interaction information from just the expression profiles, generate individual miRNA and mRNA clusters, and then merge them to form modules.

**Figure 5:**
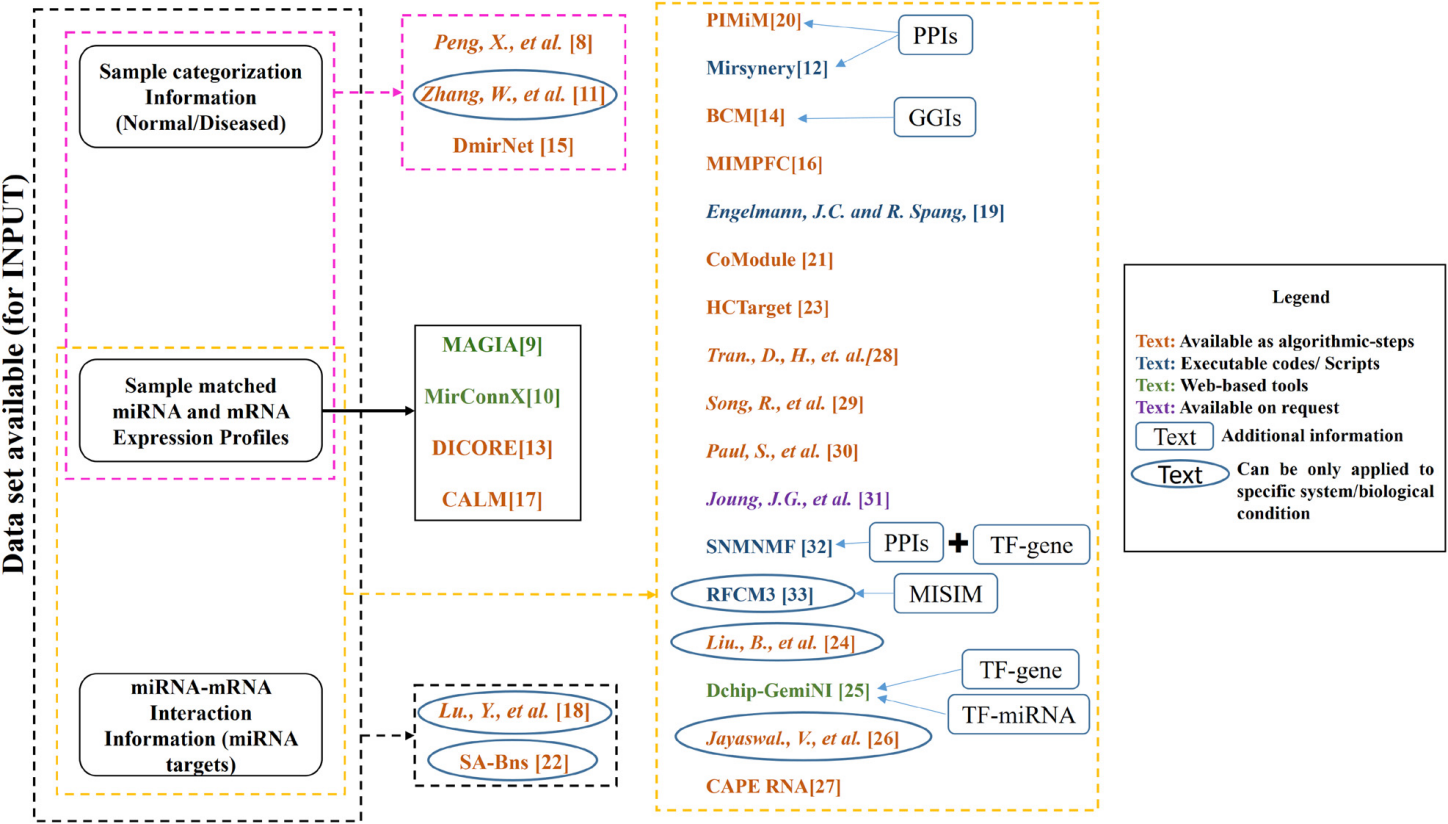
Flowchart for the selection of suitable tool as per the input data available.

The user has to be very careful while choosing the suitable method for their study. The choice of method depends on the amount and type of input data available and the biological question under investigation. A flowchart is presented in Figure 5 to guide the users for the same. For example, if someone needs to identify MRMs by incorporating miRNA-target information and PPIs along with the expression profiles, they can go for Mirsynergy [12] or PIMiM [20], and if they also want to incorporate TF-gene information, then they can go for SNMNMF [32]. There are several options available, if a person is only interested in using expression profiles to avoid incorporation of false positive interactions coming from sequence-based interaction databases. Additional information like GGIs, PPIs, miRNA-targets, MISIM and others can be beneficial if the interaction information for most of the miRNAs and mRNAs/genes present in the expression profiles is available. For a study where normal and diseased samples are available performing a prior differential expression analysis is preferred and incorporation of disease specific interactions provides better results. Methods like DmirNet [15] and Peng X. et al. [8] can be used in these scenarios.

As pointed out in Figure 4, there are three algorithmic steps for MRMs identification, (1) calculation of association between miRNAs and mRNAs from expression profiles, (2) data integration in the form of miRNA–target interaction, and (3) bi-clustering of the final interaction matrix or network. There can be one extra step where other additional information can be added. Therefore, users have the opportunity to mix and match these steps based on data availability and the mathematical model they want to use. Suppose a user wants to calculate the association between miRNAs and mRNAs using MI and not correlation and also have no MISIM [70] information for generating final MRMs as par the RFCM^3^ [33] method. He/She can use the concept of synergy score used in Mirsynergy [12] and compute the association between multiple miRNAs. A single tool/method is sufficient for MRMs identification if similar input data are chosen, but a user can also, combine the results of a few tools for a single analysis as explained above. Such combination requires through mathematical understanding and good coding skills in order to alter the original algorithm. Also for some of the methods, only mathematical details are available despite working tools hence working with them is difficult.

Some of the methods discussed here are freely available; some of them are available on request, whereas most are only available as algorithmic-steps. Hence working with them is difficult. Such methods provide little help to the end-users but are helpful for the algorithm developers in applying various mathematical models. The web-interface based methods are easy to use and are available with proper documentation. Whereas knowledge of respective programming languages is required for using the methods for which scripts are available. All such information is provided in Table 1 and the working links are provided at the point of method description (if available). Time complexity is only discussed here if it is presented by the authors of respective methods, as benchmarking is beyond the scope of this review. Time consumption depends on the number of miRNAs and mRNAs available in the input data, mathematical complexity, and the programming platform used. Methods that require incorporation of additional information requires database mining which is a time-consuming task.

## 4 Conclusions

Several efforts have been made to understand the scrupulous regulatory functions of miRNAs based on miRNA regulatory modules. However, it is still a challenge because of the complex nature of combinatorial and cooperative mechanisms between miRNAs and genes. Recently, the opportunity for identification of condition-specific MRMs has increased drastically because of the availability of same sampled expression profiles. Further, incorporation of prior target binding information and GGIs adds extra knowledge to identify MRMs. Whereas, some researchers have suggested that to avoid bias, incorporation of such information should be avoided. This review attempts to summarize the recent progress in the computational methods and tools applied for the identification of MRMs. 26 strategies that use paired mi/mRNA samples to detect functional MRMs have been surveyed. The methods discussed here take the help of predicted miRNA–mRNA targets, GGIs, TF-miRNA–gene interaction, MISIM, and gene ontology-based semantic similarity along with the expression profiles at different stages.

Most of the methods discussed here are model-dependent, and their efficiency in identifying MRMs depends on the selection and quality of the data used in their development. They also use a variety of aspects of miRNA–mRNA interactions available over time. Therefore, making a comparison between them would be a biased attempt. This review might help the readers easily identifying appropriate steps to be followed for their study. Although the reviewed methods cannot be compared directly. There is a scope that they can complement and enhance each other’s functionality if combined. The methodologies discussed in this study will help the users get an in-depth understanding of the MRMs; simultaneously, will help the algorithm developers develop more effective tools.

## References

[1] Calin GA, Ferracin M, Cimmino A, Leva GD, Shimizu M, Wojcik SE (2005). A MicroRNA signature associated with prognosis and progression in chronic lymphocytic leukemia. N Engl J Med.

[2] Bentwich I, Avniel A, Karov Y, Aharonov R, Gilad S, Barad O (2005). Identification of hundreds of conserved and nonconserved human microRNAs. Nat Genet.

[3] Jones SG, Saini HK, Dongen SV, Enright AJ (2008). Mirbase: tools for microRNA genomics. Nucleic Acids Res.

[4] Hatzigeorgiou AG (2007). Same computational analysis, different miRNA target predictions. Nat Methods.

[5] Krek A, Grun D, Poy MN, Wolf R, Rosenberg L, Epstein EJ (2005). Combinatorial microRNA target predictions. Nat Genet.

[6] Croce CM (2009). Causes and consequences of microRNA dysregulation in cancer. Nat Rev Genet.

[7] Bartel DP (2009). MicroRNAs: target recognition and regulatory functions. Cell.

[8] Peng X, Li Y, Walters KA, Rosenzweig ER, Lederer SL, Aicher LD (2009). Computational identification of hepatitis C virus associated microRNA-mRNA regulatory modules in human livers. BMC Genom.

[9] Sales G, Coppe A, Bisognin A, Biasiolo M, Bortoluzzi S, Romualdi C (2010). MAGIA, a web-based tool for miRNA and genes integrated analysis. Nucleic Acids Res.

[10] Huang GT, Athanassiou C, Benos PV (2011). mirConnX: condition-specific mRNA-microRNA network integrator. Nucleic Acids Res.

[11] Zhang W, Edwards A, Fan W, Flemington EK, Zhang K (2012). miRNA-mRNA correlation-network modules in human prostate cancer and the differences between primary and metastatic tumor subtypes. PLoS One.

[12] Yue L, Cheng L, Ka-Chun W, Jiawei L, Zhaolei Z (2014). Mirsynergy: detecting synergistic miRNA regulatory modules by overlapping neighbourhood expansion. Bioinformatics.

[13] Karim SMM, Liu L, Le TD, Li J (2016). Identification of miRNA-mRNA regulatory modules by exploring collective group relationships. BMC Genom.

[14] Liang C, Li Y, Luo J (2018). A novel method to detect functional microRNA regulatory modules by bicliques merging. IEEE ACM Trans Comput Biol Bioinf.

[15] Lee M, Lee H (2016). DMirNet: inferring direct microRNA-mRNA association networks. BMC Syst Biol.

[16] Luo D, Wang SL, Fang J, Zhang W (2018). MIMPFC: identifying miRNA-mRNA regulatory modules by combining phase-only correlation and improved rough-fuzzy clustering. J Bioinf Comput Biol.

[17] Luo J, Huang W, Cao B (2018). A novel approach to identify the miRNA-mRNA causal regulatory modules in cancer. IEEE ACM Trans Comput Biol Bioinf.

[18] Lu Y, Zhou Y, Qu W, Deng M, Zhang C (2011). A lasso regression model for the construction of microRNA-target regulatory networks. Bioinformatics.

[19] Engelmann JC, Spang R (2012). A least angle regression model for the prediction of canonical and non-canonical miRNA-mRNA interactions. PLoS One.

[20] Le HS, Joseph ZB (2013). Integrating sequence, expression and interaction data to determine condition-specific miRNA regulation. Bioinformatics.

[21] Luo J, Pan C, Xiang G, Yin Y (2019). A novel cluster-based computational method to identify miRNA regulatory modules. IEEE ACM Trans Comput Biol Bioinf.

[22] Liu B, Li J, Tsykin A, Liu L, Gaur AB, Goodall GJ (2009). Exploring complex miRNA-mRNA interactions with bayesian networks by splitting-averaging strategy. BMC Bioinf.

[23] Su N, Qian M, Deng M (2009). Integrative approaches for microRNA target prediction: combining sequence information and the paired mRNA and miRNA expression profiles. BMC Bioinf.

[24] Liu B, Liu L, Tsykin A, Goodall GJ, Green JE, Zhu M (2010). Identifying functional miRNA-mRNA regulatory modules with correspondence latent dirichlet allocation. Bioinformatics.

[25] Yan Z, Shah PK, Amin SB, Samur MK, Huang N, Wang X (2012). Integrative analysis of gene and miRNA expression profiles with transcription factor-miRNA feed-forward loops identifies regulators in human cancers. Nucleic Acids Res.

[26] Jayaswal V, Lutherborrow M, Ma DD, Yang YH (2011). Identification of microRNA-mRNA modules using microarray data. BMC Genom.

[27] Hecker N, Stephan C, Mollenkopf HJ, Jung K, Preissner R, Meyer HA (2013). A new algorithm for integrated analysis of miRNA-mRNA interactions based on individual classification reveals insights into bladder cancer. PLoS One.

[28] Tran DH, Satou K, Ho TB (2008). Finding MicroRNA regulatory modules in human genome using rule induction. BMC Bioinf.

[29] Song R, Liu Q, Liu T, Li J (2015). Connecting rules from paired miRNA and mRNA expression data sets of HCV patients to detect both inverse and positive regulatory relationships. BMC Genom.

[30] Paul S, Lakatos P, Hartmann A, Stock RS, Vera J (2017). Identification of miRNA-mRNA modules in colorectal cancer using rough hypercuboid based supervised clustering. Sci Rep.

[31] Joung JG, Hwang KB, Nam JW, Kim SJ, Zhang BT (2007). Discovery of microRNA-mRNA modules via population-based probabilistic learning. Bioinformatics.

[32] Zhang S, Li Q, Liu J, Zhou XJ (2011). A novel computational framework for simultaneous integration of multiple types of genomic data to identify microRNA-gene regulatory modules. Bioinformatics.

[33] Paul S, Madhumita (2020). RFCM 3: computational method for identification of miRNA-mRNA regulatory modules in cervical cancer. IEEE ACM Trans Comput Biol Bioinf.

[34] Lim LP, Lau NC, Garrett-Engele P, Grimson A, Schelter JM, Castle J (2005). Microarray analysis shows that some microRNAs downregulate large numbers of target mRNAs. Nature.

[35] Place RF, Li LC, Pookot D, EJN EJ, Dahiya R (2008). MicroRNA-373 induces expression of genes with complementary promoter sequences. Proc Natl Acad Sci Unit States Am.

[36] Ørom UA, Nielsen FC, Lund AH (2008). MicroRNA-10a binds the 5 UTR of ribosomal protein mRNAs and enhances their translation. Mol Cell.

[37] Nazarov PV, Reinsbach SE, Muller A, Nicot N, Philippidou D, Vallar L (2013). Interplay of microRNAs, transcription factors and target genes: linking dynamic expression changes to function. Nucleic Acids Res.

[38] West DB (2001). Introduction to graph theory upper saddle river. J Mach Learn Res.

[39] Nepusz T, Yu H, Paccanaro A (2012). Detecting overlapping protein complexes in protein-protein interaction networks. Nat Methods.

[40] Hotelling H (1936). Relations between two sets of variants. Biometrika.

[41] Schafer J, Strimmer K (2005). A shrinkage approach to large-scale covariance matrix estimation and implications for functional genomics. Stat Appl Genet Mol Biol.

[42] Peng J, Wang P, Zhou N, Zhu J (2009). Partial correlation estimation by joint Sparse regression models. J Am Stat Assoc.

[43] Feizi S, Marbach D, Medard M, Kellis M (2013). Network deconvolution as a general method to distinguish direct dependencies in networks. Nat Biotechnol.

[44] Horner JL, Gianino PD (1984). Phase-only matched filtering. Bioinformatics.

[45] Karim SM, Liu L, Li J (2014). Discovering Collective Group Relationships.

[46] Maathuis MH, Colombo D, Kalisch M, Buhlmann P (2010). Predicting causal effects in large-scale systems from observational data. Nat Methods.

[47] Maathuis MH, Colombo D, Kalisch M, Buhlmann P (2008). Estimating high-dimensional intervention effects from observational data. Ann Stat.

[48] Spirtes P, Glymour C, Scheines R (2003). Causation, prediction, and search, second edition. Stat Med.

[49] Perl J (2000). Causality: models, reasoning, and inference. Econom Theor.

[50] Yu G, Li F, Qin Y, Bo X, Wu Y, Wang S (2010). Gosemsim: an R package for measuring semantic similarity among GO terms and gene products. Bioinformatics.

[51] Friedman N, Linial M, Nachman I, Pe’er D (2000). Using bayesian networks to analyze expression data. J Comput Biol.

[52] Davison AC, Hinkley DV (1997). Bootstrap Methods and their Application Cambridge Series in Statistical and Probabilistic Mathematics.

[53] John B, Enright AJ, Aravin A, Tuschl T, Sander C, Marks DS (2005). Human microRNA targets. PLoS Biol.

[54] Lewis BP, Burge CB, Bartel DP (2005). Conserved seed pairing, often flanked by adenosines, indicates that thousands of human genes are microRNA targets. Cell.

[55] Megraw M, Sethupathy P, Corda B, Hatzigeorgiou AG (2007). miRGen: a database for the study of animal microRNA genomic organization and function. Nucleic Acids Res.

[56] Bandyopadhyay S, Mitra R (2009). TargetMiner: microRNA target prediction with systematic identification of tissue-specific negative examples. Bioinformatics.

[57] Lewis BP, Shih IH, Jones-Rhoades MW, Bartel DP, Burge CB (2003). Prediction of mammalian microRNA targets. Cell.

[58] Blei DM, Jordan MI (2003). Modeling annotated data. ..

[59] Pham TH, Clemente JC, Satou K, Ho TB (2005). Computational discovery of transcriptional regulatory rules. Bioinformatics.

[60] Lavrac N, Kavsek B, Flach P, Todorovski L (2004). Subgroup discovery with CN2-SD. J Mach Learn Res.

[61] Clark P, Nibblet T (1989). The CN2 induction algorithm. J Mach Learn Res.

[62] Paul S, Vera J (2015). Rough hypercuboid based supervised clustering of miRNAs. Mol Biosyst.

[63] Quinlan JR (1994). C4. 5: Programs for machine learning.

[64] Han J, Kamber M (2006). Data mining: concepts and techniques.

[65] Baluja S (1994). Population-Based Incremental Learning: A Method for Integrating Genetic Search Based Function Optimization and Competitive Learning.

[66] Larranaga P, Lozano JA (2002). Estimation of Distribution Algorithms: A New Tool for Evolutionary Computation.

[67] Zhang BT., Ghosh A, Tsutsui S (2003). A unified bayesian framework for evolutionary learning and optimization. Advances in evolutionary computing. Natural computing series.

[68] Madeira SC, Oliveira AL (2004). Biclustering algorithms for biological data analysis: a survey. IEEE ACM Trans Comput Biol Bioinf.

[69] Lee DD, Seung HS (2001). Algorithms for non-negative matrix factorization. Adv Neural Inf Process Syst.

[70] Wang D, Wang J, Lu M, Song F, Cui Q (2010). Inferring the human MicroRNA functional similarity and functional network based on MicroRNA-associated diseases. Bioinformatics.

[71] Maji P (2009). f-information measures for efficient selection of discriminative genes from microarray data. IEEE Trans Syst Man Cybern C Appl Rev.

[72] Huang DW, Sherman BT, Lempicki RA (2009). Systematic and integrative analysis of large gene lists using DAVID bioinformatics resources. Nat Protoc.

[73] Huang DW, Sherman BT, Lempicki RA (2009). Bioinformatics enrichment tools: paths toward the comprehensive functional analysis of large gene lists. Nucleic Acids Res.

